# SIRT6 in Vascular Diseases, from Bench to Bedside

**DOI:** 10.14336/AD.2021.1204

**Published:** 2022-07-11

**Authors:** Si-Chong Ren, Xiangqi Chen, Hui Gong, Han Wang, Chuan Wu, Pei-Heng Li, Xiao-Feng Chen, Jia-Hua Qu, Xiaoqiang Tang

**Affiliations:** ^1^Key Laboratory of Birth Defects and Related Diseases of Women and Children of MOE, State Key Laboratory of Biotherapy, West China Second University Hospital, Sichuan University, Chengdu, China.; ^2^Department of Nephrology, Clinical Medical College and The First Affiliated Hospital of Chengdu Medical College, Chengdu, China.; ^3^The Lab of Aging Research, National Clinical Research Center for Geriatrics, State Key Laboratory of Biotherapy, West China Hospital, Sichuan University, Chengdu, China.; ^4^Department of Thyroid and Parathyroid Surgery, Laboratory of Thyroid and Parathyroid Disease, Frontiers Science Center for Disease-related Molecular Network, West China Hospital, Sichuan University, Chengdu, Sichuan, China.; ^5^Department of Biochemistry and Molecular Biology, Basic Medical College, Chengdu University of Traditional Chinese Medicine, Chengdu, China.; ^6^Laboratory of Cardiovascular Science, Intramural Research Program, National Institute on Aging, National Institutes of Health, Baltimore, Maryland, USA

**Keywords:** aging, sirtuin, sirt6, angiogenesis, vascular disease, activator

## Abstract

Aging is a key risk factor for angiogenic dysfunction and cardiovascular diseases, including heart failure, hypertension, atherosclerosis, diabetes, and stroke. Members of the NAD^+^-dependent class III histone deacetylase family, sirtuins, are conserved regulators of aging and cardiovascular and cerebrovascular diseases. The sirtuin SIRT6 is predominantly located in the nucleus and shows deacetylase activity for acetylated histone 3 lysine 56 and lysine 9 as well as for some non-histone proteins. Over the past decade, experimental analyses in rodents and non-human primates have demonstrated the critical role of SIRT6 in extending lifespan. Recent studies highlighted the pleiotropic protective actions of SIRT6 in angiogenesis and cardiovascular diseases, including atherosclerosis, hypertension, heart failure, and stroke. Mechanistically, SIRT6 participates in vascular diseases *via* epigenetic regulation of endothelial cells, vascular smooth muscle cells, and immune cells. Importantly, SIRT6 activators (e.g., MDL-800/MDL-811) have provided therapeutic value for treating age-related vascular disorders. Here, we summarized the roles of sirtuins in cardiovascular diseases; reviewed recent advances in the understanding of SIRT6 in vascular biology, cardiovascular aging, and diseases; highlighted its therapeutic potential; and discussed future perspectives.

## 1. Introduction

The increasing aging population has created a huge social burden as it consumes large amounts of human, economic, and medical resources. Various diseases are correlated with aging, including cardiovascular diseases, stroke, cancer, and chronic obstructive pulmonary disease [1,2]. For instance, aging is a key risk factor for cardiovascular and cerebrovascular diseases and the vascular complications of currently widespread SARS-CoV-2 infections [3]. Anti-aging drugs such as metformin and rapamycin have shown great potential for treating aging-related cardiac diseases and vascular diseases including hypertension, atherosclerosis, and stroke in preclinical animal models and humans [4-6]. Understanding the functions and mechanisms of aging and its regulators is critical for designing strategies to treat aging-related cardiovascular diseases [7,8].

In recent decades, numerous pivotal regulators of aging have been identified, including sirtuins, mammalian target of rapamycin, insulin-like growth factor (IGF), AMP-activated protein kinase, and forkhead box O (FoxO) transcription factor family [9-11]. These regulators critically participate in aging-related cardiovascular diseases, including heart failure, atherosclerosis, stroke, hypertension, and arterial aneurysms [7,12,13].

## 2. Sirtuins in Aging and Cardiovascular Diseases

**Figure 1.**
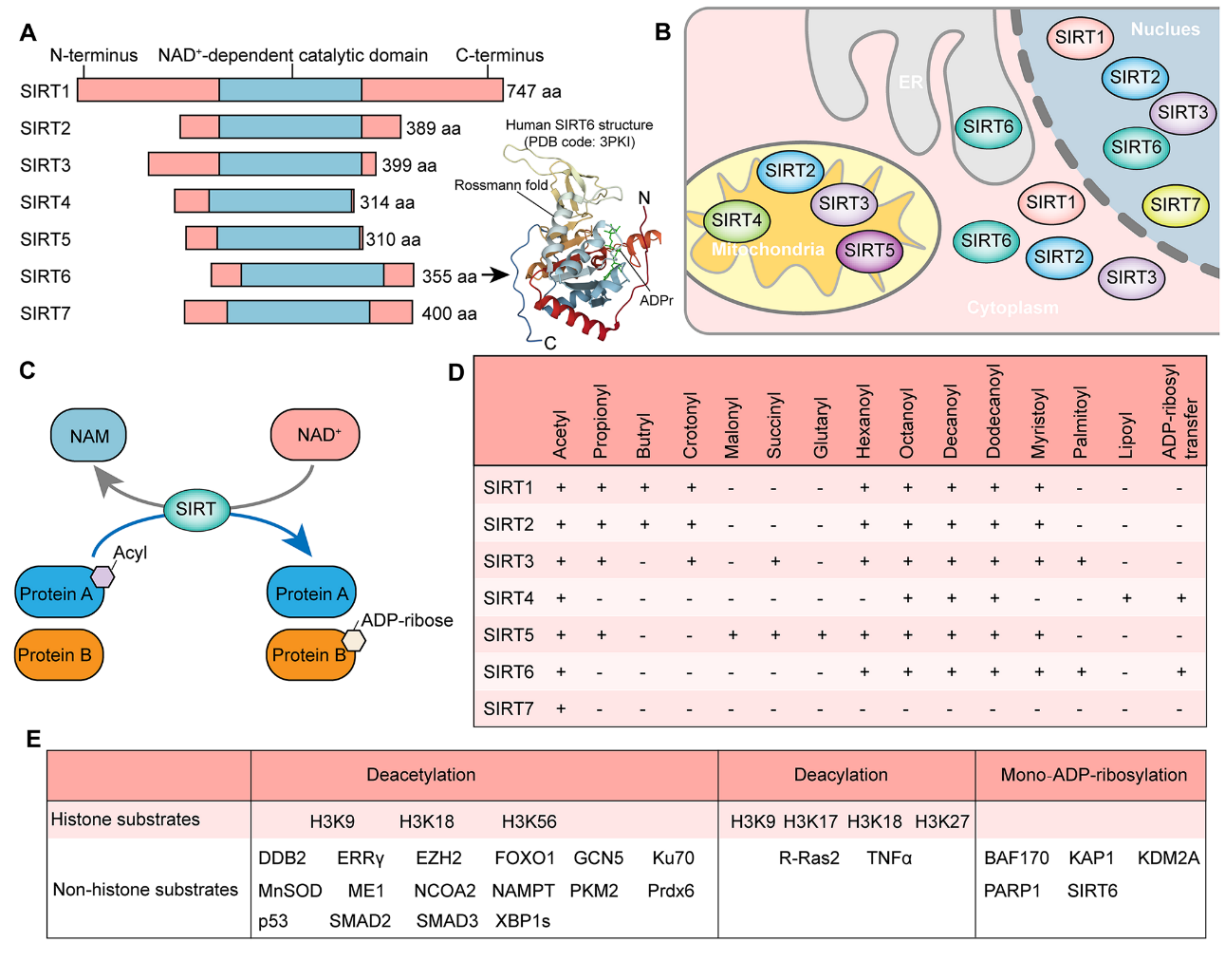
**Basic information about Sirtuins**. (**A**) Structures of Sirtuin members (left) and key domains of SIRT6 (PDB code: 3PKI). (**B**) Subcellular locations of Sirtuin members. ER, endoplasmic reticulum. (**C**) The enzymatic activity model catalyzed by Sirtuin members. NAD, nicotinamide adenine dinucleotide; NAM, nicotinamide. (**D**) Enzyme activity of Sirtuins for deacylation and ADP-ribosylation. (**E**) Substrates of SIRT6. DDB2, Damage specific DNA binding protein 2; ERRγ, Estrogen-related receptor γ; EZH2, Enhancer Of Zeste 2 polycomb repressive complex 2 subunit; FOXO1, Forkhead Box O1; GCN5, General control nonderepressible 5; Ku70, Ku autoantigen P70 subunit; MnSOD, Manganese-containing superoxide dismutase; ME1, Malic enzyme 1; NCOA2, Nuclear receptor coactivator 2; NAMPT, Nicotinamide phosphoribosyltransferase; PKM2, Pyruvate kinase M2; Prdx6, Peroxiredoxin 6; SMAD2, SMAD family member 2; XBP1s, Spliced form of X-box binding protein 1; R-Ras2, RAS related 2; TNFα, Tumor necrosis factor-alpha; BAF170, BRG1-associated factor 170; KAP1, KRAB domain-associated protein 1; KDM2A, Lysine demethylase 2A; PARP1, Poly (ADP-ribose) polymerase 1.

The sirtuin family comprises NAD^+^-dependent class III histone deacetylases with deacetylase and ADP-ribose transferase activities [13-15]. In mammalian cells, the sirtuin family consists of seven members (SIRT1-SIRT7), with SIRT1, SIRT6, and SIRT7 in the nucleus; SIRT2 in the cytoplasm; and SIRT3-5 in the mitochondria predominantly [13] (Fig. 1). Interestingly, sirtuins also shuttle across subcellular locations in response to stress. For instance, most SIRT6 is found in the nucleus, where it shows deacetylase activity for acetylated histone 3 lysine 56 (H3K56ac) and lysine 9 (H3K9ac). SIRT6 also deacetylates non-histone proteins in the nucleus and cytoplasm [16]. In addition, it also exhibits ADP-ribose transferase activity and can hydrolyze long-chain fatty acyl lysine such as myristoyl within the endoplasmic reticulum [17,18]. Thus, SIRT6 is a multifunctional epigenetic enzyme (Fig. 1E).

**Table 1:** Effects of Sirtuin knockout (KO)/transgene (TG) on animal lifespan and cardiac homeostasis.

	Genetic Alteration	Cells Targeted	Lifespan	Cardiac phenotype	Ref.
**SIRT1**	KO	ALL	Reduced	Cardiac developmental defect and cardiomyocyte apoptosis	[21]
	KO	Cardiomyocytes	NA	Augmented ischemic injury	[114]
	KO	Cardiomyocytes	Reduced	Cardiac abnormalities, arrhythmia-related premature death	[26]
	TG	Cardiomyocytes	NA	Reduced cardiomyocyte toxicity induced by chemical injury	[115]
	TG of mutant SIRT1	Cardiomyocytes	Reduced	Dilated cardiomyopathy and cardiomyocyte apoptosis	[19]
**SIRT2**	KO	ALL	NA	Aging-related cardiac fibrosis and cardiac hypertrophy	[25]
	TG	Cardiomyocytes	NA	Repressed Ang II-induced cardiac hypertrophy	[25]
**SIRT3**	KO	ALL	NA	Spontaneous cardiac hypertrophy	[33]
	TG	Cardiomyocytes	NA	Repressed cardiac hypertrophy induced by pressure overload and aging	[33,116]
**SIRT4**	KO	ALL	NA	Repressed Ang II-induced cardiac hypertrophy and fibrosis	[35]
	TG	Cardiomyocytes	NA	Promoted Ang II-induced cardiac hypertrophy	[35]
SIRT5	KO		NA	Promoted Ang II-induced cardiac hypertrophy, augmented ischemic injury	[44,117]
**SIRT6**	KO	ALL	Reduced	Spontaneous cardiac hypertrophy	[46]
	KO	Cardiomyocytes	NA	Increased cardiac hypertrophy induced by pressure-overload	[46]
	TG	Cardiomyocytes	NA	Repressed cardiac hypertrophy induced by pressure-overload	[46]
**SIRT7**	KO	ALL	Reduced	Spontaneous inflammatory cardiomyopathy	[20]
	KO	Cardiomyocytes	NA	Increased cardiac hypertrophy induced by pressure-overload	[118]

KO, knockout; TG, transgene; Ang II, angiotensin II; N/A, not available.

Sirtuins are enzymes that slow the process of aging and aging-related disorders across species. Genetic depletion of SIRT1, SIRT6, and SIRT7 affects the development and reduces the lifespan of mice, accompanied by cardiovascular defects [18-21]. Although the effects of SIRT2 on lifespan have not been investigated, studies from our lab and others have highlighted the role of SIRT2 in preventing aging-related cardiovascular remodeling, reproductive aging, aging-associated chronic inflammation, and hematopoietic stem cell aging [22-25]. However, the effects of mitochondrial sirtuin members on lifespan in animals remain unclear. Given the roles of mitochondrial sirtuins in key metabolic and cellular processes that are directly linked to aging, their activities should be further investigated.

The roles of sirtuins in cardiovascular diseases have been widely studied over the past decade. In general, sirtuin family members, except for SIRT4, are cardioprotective factors (Table 1). SIRT1 has been shown to protect the heart from aging-related cardiac remodeling, arrhythmia, and ischemic injury [26-29]. Our previous findings revealed that SIRT1 participates in cardiac development by deacetylating P53 and NKX2.5 [19,30]. We also showed that SIRT2 was a critical factor preventing aging-related cardiac remodeling *via* activation of liver kinase B1-AMP-activated protein kinase signaling and contributes to metformin-mediated anti-hypertrophic effects [25]. The cardioprotective role of SIRT2 was validated in follow-up studies [31,32]. SIRT3 and SIRT4 regulate cardiac remodeling by targeting mitochondrial metabolism and reactive oxide species (ROS) homeostasis. SIRT3 represses aging-related and stress-induced cardiac hypertrophy and fibrosis by deacetylating FOXO3 and manganese-containing superoxide dismutase (MnSOD) to reduce the levels of mitochondrial ROS [33,34]. Furthermore, we previously showed that SIRT4 inhibited the SIRT3-MnSOD interaction to repress MnSOD deacetylation and its antioxidative activity, thus indicating its pro-hypertrophic role [35].

SIRT3-mediated metabolic homeostasis in mitochondria also contributes to its cardioprotective functions [36,37]. Interestingly, a recent report revealed that SIRT3 was shuttled into the nucleus to inhibit FOS via histone H3 deacetylation and subsequently prevented cardiac fibrosis and inflammation [38]. Mitochondrial sirtuins also regulate short-chain lysine acylations to participate in cardiovascular biology [39,40]. For instance, SIRT3 deacetylated ECHS1 [41], which repressed cardiac hypertrophy by inhibiting histone crotonylation [42]. Additionally, SIRT5 maintains metabolic homeostasis by regulating mitochondrial succinylation to preserve cardiac function and increase the survival of animals in response to cardiac pressure overload [43,44]. SIRT6 also regulates metabolism to participate in cardiac remodeling. SIRT6 represses IGF-AKT signaling to reduce cardiac aging and hypertrophy while activating FOXO3 to reduce injury induced by ischemia [45,46].

Interestingly, sirtuins play important roles in cardiac tissues in a deacetylase-independent manner. For instance, we observed that the effects of SIRT4 on MnSOD and mitochondrial ROS in hypertrophic cardiomyocytes did not rely on its enzymatic activity [35]. Moreover, the deacetylase-independent function of SIRT6 is coupled with the transcription factor GATA-binding protein 4 and epigenetic activation to prevent cardiomyocyte apoptosis induced by doxorubicin [47]. Taken together, sirtuins function as cardioprotective factors, except for SIRT4 and SIRT1-3, which have been shown to inhibit cardiac aging.

**Table 2:** Roles of Sirtuin knockout (KO)/transgene (TG) on vascular homeostasis.

	Genetic Alteration	Cells Targeted	Vascular Phenotype	Ref.
**SIRT1**	KO	Endothelial cells	Promoted vascular aging with reduced muscle capillary, nephrosclerosis, and atherosclerosis	[119,120] [52,121]
	KO	Macrophages	Promoted Ang II-induced abdominal aortic aneurysm	[52]
	KO	VSMC	Promoted abdominal aortic aneurysm;	[48,53]
	TG	Endothelial cells	Inhibited hyperglycemia-induced endothelial dysfunction and atherosclerosis	[49,51]
	TG	VSMC	Inhibited abdominal aortic aneurysm, injury-induced neointima formation, and diet-induced aortic stiffness	[50,53,122]
**SIRT3**	KO	ALL	Spontaneous pulmonary arterial hypertension (PAH); PAH associated with HFpEF; promoted Ang II-induced hypertension and accelerated arterial thrombosis	[56,58,59,123,124]
	TG	ALL	Attenuated Ang II/deoxycorticosterone acetate-salt induced hypertension	[57]
**SIRT5**	KO	ALL	Blunted arterial thrombosis	[125]
	TG	ALL	Accelerated arterial thrombus formation	[125]
**SIRT6**	Heterozygote	ALL	Promoted atherosclerosis	[78,84]
	KO	Endothelial cells	Exacerbated hypertension and complications; enhanced atherosclerosis, stroke, and vascular aging.	[77,79,90,126]
	TG	VSMC	Reduced atherosclerosis	[88]
**SIRT7**	KO	ALL	Enhanced neointimal formation	[127]
	TG	VSMC	Attenuated neointimal formation	[127]
	TG	Endothelial cells	Extended lifespan in Hutchinson-Gilford progeria syndrome	[128]

KO, knockout; TG, transgene; VSMC, vascular smooth muscle cells; HFpEF, heart failure with preserved ejection fraction.

The roles of SIRT1 and SIRT3 in vascular biology have been widely studied (Table 2). SIRT1 is a well-known anti-aging factor in vascular diseases, and our previous studies demonstrated the role of SIRT1 in preventing atherosclerosis, hypertension, and abdominal aortic aneurysm [3,48-54]. In vascular tissues, SIRT1 is a multifunctional protective factor that reduces the senescence of vascular smooth muscle cells (VSMCs), regulates M2 macrophage polarization, and prevents endothelial dysfunction and senescence, which have been discussed in previous reviews by our team and others [3,13,55]. SIRT3 was shown to play a role in hypertension and pulmonary arterial hypertension (PAH). Loss of SIRT3 induced a spontaneous PAH phenotype in mice, which was accompanied by metabolic dysfunction and VSMC hyperproliferation [56]. This finding was validated in humans; patients with single-nucleotide polymorphisms in SIRT3 showed an increased risk of PAH [56]. SIRT3 also represses stress-induced hypertension by regulating MnSOD hypoacetylation and ROS homeostasis in endothelial cells and by repressing endothelial-to-mesenchymal transition [57-59]. For a long time, the *in vivo*roles of other sirtuins in vascular biology and aging-related vascular diseases remained largely unknown. Over the past two years, the understanding of SIRT6 in vascular diseases has achieved remarkable progress.

## 3. SIRT6 Regulation of Aging

**Figure 2.**
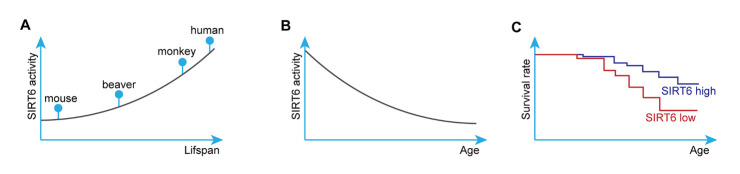
**SIRT6 function in regulating aging**. (**A**) SIRT6 activity is higher in long-lived species. SIRT6 is responsible for more efficient DNA double-strand break repair in long-lived species. (**B**) SIRT6 activity declines with aging in primates and rodents. (**C**) SIRT6 high expression expands lifespan in mice.

Among the sirtuin family members, SIRT6 is a key regulator of genome stability, stemness, and aging. SIRT6 deficiency reduces the lifespan of mice [18,60,61]. In 2012, Kanfi et al. showed that transgenic mice overexpressing SIRT6 had a remarkably longer lifespan than their wild-type littermates, suggesting that SIRT6 has important therapeutic potential in aging-related diseases [62]. In 2018, using a genome editing strategy, Zhang et al. showed that SIRT6 is a pivotal regulator of development and lifespan in non-human primates [63], revealing an orchestrator role for SIRT6 in mammalian aging. Interestingly, SIRT6 is more responsible for efficient DNA double-strand break repair [64], a conserved mechanism for preventing cell senescence and aging, in long-lived species (Fig. 2A), suggesting that SIRT6 activity is increased in long-lived species and that the activity of this enzyme is associated with lifespan. Although the activity of SIRT6 decreases with age [65,66] (Fig. 2B), a clinical study implicated a low level of SIRT6 methylation (high SIRT6 expression) as a protective factor in the longevity of Chinese people [67]. In rodents, genetic overexpression of SIRT6 partially prolongs lifespan by regulating IGF-AKT signaling, contributing to SIRT6 function in preventing cardiac aging and heart failure (Fig. 2C) [46,62]. Thus, SIRT6 is a critical protein regulating lifespan, making this protein a hotspot in the field of aging-related diseases, including vascular diseases.

The role of stem cells in aging is critical, and some studies have focused on the functions and mechanisms underlying SIRT6 in stem cells. For instance, Pan et al. [68] reported that SIRT6 protected against oxidative stress by activating NF-E2-related factor 2 in human mesenchymal stem cells. A breakthrough study led by Wang and Ju showed that SIRT6 controls the homeostasis of hematopoietic stem cells in mice through epigenetic regulation of WNT signaling by deacetylating H3K56ac [69], highlighting the critical role of SIRT6 in maintaining the stemness of these cells. Our work revealed important roles for SIRT1 and SIRT6 in mouse somatic reprogramming and pluripotency maintenance [70,71]. Thus, SIRT6 controls stemness. Notably, the SIRT6 activator MDL-800 was recently shown to improve the genome stability and pluripotency of aged murine-derived induced pluripotent stem cells [72]. The ability of SIRT6 to regulate pluripotency and differentiation depends mainly on its effects on histones and chromatin [69,73]. For instance, SIRT6 regulates Tet-mediated production of 5hmC to serve as a chromatin regulator that safeguards the balance between pluripotency and differentiation [74]. In non-human primates, genetic loss of SIRT6 delays neuronal differentiation and shortens lifespan [63]. These studies suggest that SIRT6 regulates stemness (anti-senescence) and physiological differentiation to reduce aging and aging-related disorders. Further studies are needed to validate whether SIRT6 regulates aging and lifespan by controlling the senescence of stem cells, such as hematopoietic stem cells and vascular progenitor cells.

## 4. SIRT6 in Vascular Diseases

Senescence, phenotype switching, and activation of vascular cells and immune cells are hallmarks of vascular aging and contribute to vascular diseases, including atherosclerosis, hypertension, stroke, arterial aneurysm, and vascular injury in myocardial infarction. The critical role of SIRT6 in regulating aging and stem cell senescence has driven studies of SIRT6 in vascular aging and diseases. Recent studies from our lab and others revealed that SIRT6 protects against vascular aging. SIRT6 is widely expressed in vascular cells and participates in vascular biology by epigenetically regulating endothelial cells (ECs), VSMCs, and immune cells. Here, we discuss recent advances in the understanding of SIRT6 in aging-related vascular diseases, including atherosclerosis, hypertension, and ischemic stroke.

### 4.1 Atherosclerosis

**Figure 3.**
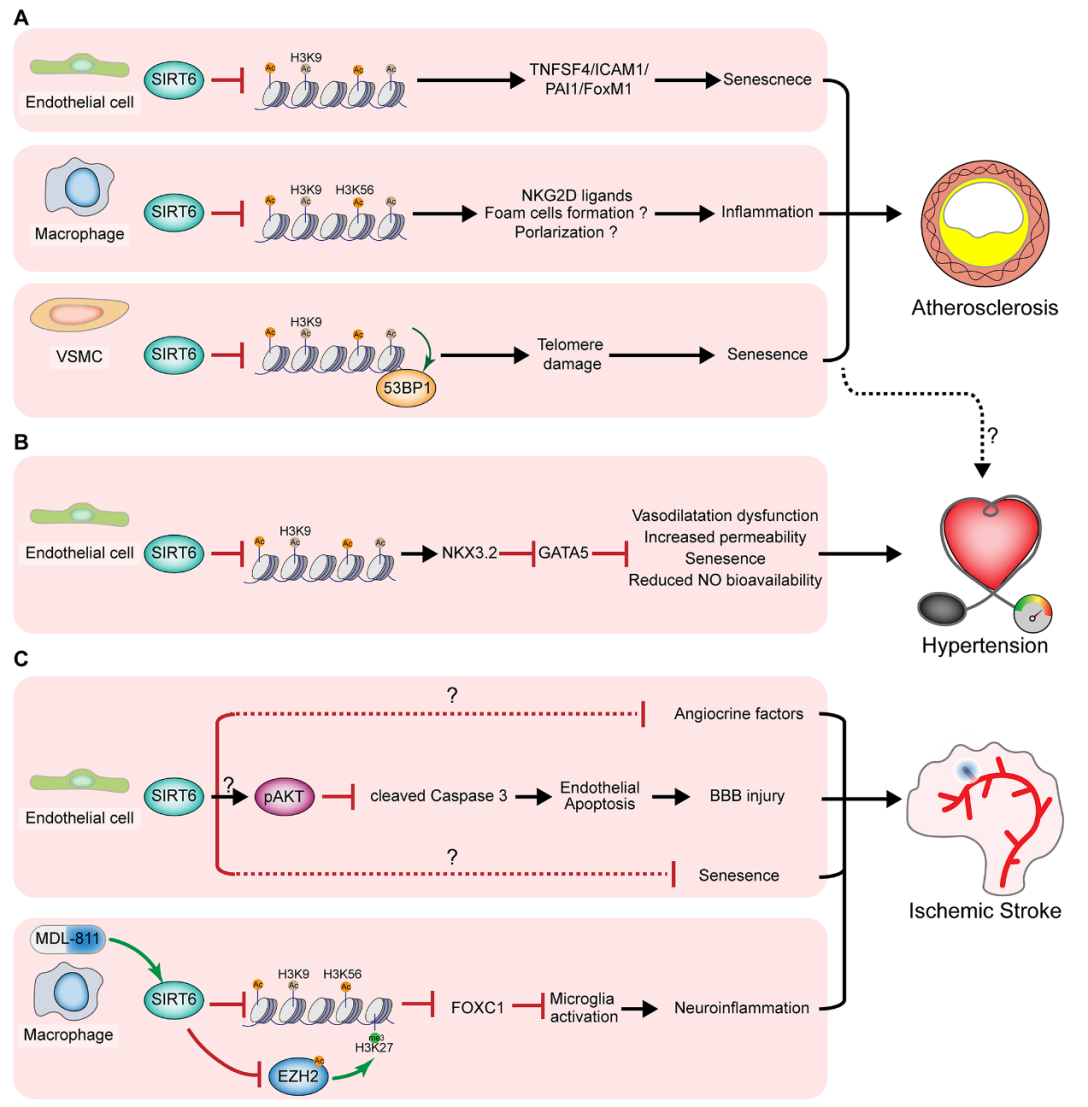
**SIRT6 function in regulating vascular disease (Central Illustration)**. (**A**) SIRT6 represses the initiation, development, and plaque instability of atherosclerosis. In endothelial cells, SIRT6 epigenetically represses the production of pro-inflammatory angiocrine factors and senescence-associated secretory phenotype, thus inhibiting endothelial dysfunction and senescence to reduce initiation and development of atherosclerosis. In macrophages, SIRT6 deacetylates H3K9ac and H3K56ac to reduce the expression of natural-killer group 2, member D (NKG2D) ligands, inhibiting the activation of immune cells and atherosclerosis development. SIRT6 also maintains the telomere integrity by deacetylating H3K9ac at the telomere and inhibiting 53BP1 binding, thus suppressing vascular smooth muscle cells (VSMCs) senescence. SIRT6 deficiency leads to VSMC senescence and plaque instability of atherosclerosis. PAI1, plasminogen activator inhibitor-1; TNFSF4, TNF superfamily member 4; FoxM1, Forkhead box protein M1; ICAM1, intercellular adhesion molecule-1. (**B**) SIRT6 suppresses hypertension. By deacetylating H3K9ac, endothelial SIRT6 inhibits NKX3.2 (NK3 homeobox 2) expression to reduce the transcription of GATA5 (GATA-binding protein 5), a transcriptional factor controlling blood pressure. Endothelial loss of SIRT6 facilitates hypertension and associated cardiorenal injury. SIRT6-mediated suppression of VSMC may also contribute to its role in preventing hypertension. (**C**) SIRT6 inhibits ischemic stroke. Endothelial loss of SIRT6 induces AKT inhibition *via* an unknown mechanism, which activates Caspase 3 to cause endothelial apoptosis and subsequent blood-brain barrier (BBB) injury and ischemic stroke. It remains unknown whether SIRT6 regulates endothelial senescence and angiocrine phenotype to participate in ischemic stroke. Chemical drug MDL-811 can activate macrophage SIRT6 and repress ischemic stroke *via* targeting histone acetylation and EZH2 activation to promote the expression of FOXC1.

Endothelial dysfunction and senescence are the initial steps in atherosclerosis development. SIRT6 is a well-known regulator of endothelial senescence. As discussed previously, SIRT6 is essential for DNA damage repair and genome stability in stem cells and plays a similar role in vascular cells. SIRT6 deficiency in human ECs increases DNA damage, the formation of telomere dysfunction-induced foci, and the fraction of senescent cells as well as reduces cell proliferation and angiogenesis capacity [75]. In human endothelial cells, SIRT6 represses the expression of senescence-associated angiocrine factors, such as plasminogen activator inhibitor-1 and TNF superfamily member 4, and that of forkhead box M1 [75-77]. In ApoE^-/-^ mice fed a high-fat diet (HFD), endothelial loss of SIRT6 promotes monocyte adhesion to ECs, impairs endothelium-dependent vasorelaxation, and facilitates atherosclerosis development [78,79]. This function of SIRT6 was partially mediated by decreases in the gene expression of TNF superfamily member 4 and forkhead box M1 through binding to and deacetylating H3K9ac at the gene promoters (Fig. 3A) [77,78]. Collectively, SIRT6 guards against endothelial dysfunction and senescence by deacetylating H3K9ac to prevent DNA damage and the senescence-associated secretory phenotype. Endothelial dysfunction is the initiation step in the development of atherosclerosis, indicating that SIRT6 participates in the early stages of the disease [80,81]. Patients with diabetic atherosclerosis showed higher endothelial SIRT6 in plaques following administration of glucagon-like peptide-1 (GLP-1) receptor agonists [82], revealing endothelial SIRT6 as a curative effect index and target for atherosclerosis treatment. Endothelial SIRT6 facilitates angiogenesis and hemorrhage of carotid plaques by inhibiting HIF-1α and ROS [83]. Thus, the anti-atherosclerotic role of SIRT6 may also rely on its function in angiogenesis. Studies are needed to determine whether SIRT6 activation in ECs can reverse established plaques.

Immune cells are essential for vascular homeostasis, aging, and disease. Macrophage activation is required for the initiation of plaque formation and plaque instability in atherosclerosis. Our findings revealed that SIRT6 in macrophages inhibited HFD-induced atherosclerosis in ApoE^-/-^ mice by reducing the levels of H3K9ac and H3K56ac. This reduction represses the expression of ligands for natural killer (NK) group 2D (NKG2D), which is critical for activating NK cells to favor atherosclerosis development and plaque instability (Fig. 3A) [84,85]. However, we did not examine the role of SIRT in polarization switching and foam cell formation, which are two crucial features of macrophages in atherosclerotic plaques. A follow-up study of bone marrow transplantation validated the important role of macrophage SIRT6 in preventing atherosclerosis in mice [86]. In addition, another follow-up study showed that SIRT6 repressed the expression of macrophage scavenger receptor 1, a receptor for oxidative low-density lipoprotein uptake and foam cell formation, in the atherosclerotic plaques of ApoE^-/-^ mice [86]. During HFD-induced insulin resistance in the liver, SIRT6 deficiency promoted M1 macrophage transformation and the inflammatory response [87], suggesting that SIRT6-mediated macrophage polarization contributes to its effects in atherosclerosis. Therefore, SIRT6 in macrophages may prevent atherosclerosis by regulating macrophage polarization and inflammation as well as foam cell formation to inhibit the initiation of atherosclerosis and plaque instability. In other types of immune cells, SIRT6 may also be necessary for preventing atherosclerosis. More detailed experiments are required to validate the effects of SIRT6 on immune cells, such as macrophages and T lymphocytes, in atherosclerotic plaques. Additionally, high-throughput strategies such as chromatin immunoprecipitation-seq may reveal the direct targets of SIRT6 in immune cells.

Generally, VSMCs undergo phenotypic switching and senescence in atherosclerotic plaques. Delaying VSMC senescence is an approach for maintaining plaque stability and reversing atherosclerosis. Grootaert et al. [88] reported that SIRT6 protected VSMCs from senescence by decreasing H3K9 acetylation levels in telomeres to maintain telomere integrity in humans and mice (Fig. 3A). In VSMCs from human and mouse plaques, SIRT6 deacetylated telomere chromatin, leading to reduced 53BP1 binding and VSMC senescence. Genetic overexpression of SIRT6 in VSMCs delayed senescence and inhibited atherosclerosis in HFD-fed ApoE^-/-^ mice. This was the first study to investigate the role of SIRT6 in VSMCs *in vivo*. A previous *in vitro*study showed that SIRT6 regulates VSMCs to switch their phenotypes from a quiescent contractile phenotype to a synthetic phenotype [89], which may also contribute to the function of SIRT6 in atherosclerosis. Thus, SIRT6 represses VSMC phenotype switching and senescence to increase plaque stability and delay the development of atherosclerosis. However, the *in vivo*study of Grootaert et al. was based on transgenic mice overexpressing SIRT6, which greatly differs from physiological and pathological conditions. Therefore, further studies using VSMC-specific knockout mice are needed to determine the vascular protective roles of endogenous VSMC SIRT6.

### 4.2 Hypertension

The critical functions of SIRT6 in regulating ECs and VSMCs also affect hypertension. In ECs, SIRT6 deacetylated H3K9ac at the promoter of Nkx3.2 (NK3 homeobox 2) and repressed its expression. Nkx3.2 is a transcription factor that controls the expression of GATA5, a novel regulator of blood pressure [90]. Endothelial overexpression of SIRT6 showed therapeutic potential in deoxycorticosterone acetate/salt-induced hypertension and related cardiorenal syndromes in mice (Fig. 3B). SIRT6 showed pleiotropic protective effects in ECs by promoting endothelium-dependent vasodilatation and vascular nitric oxide bioavailability to ameliorate endothelial senescence [90]. SIRT6 also participates in hypertensive nephropathy in humans and mice [91].

Angiotensin-converting enzyme 2 (ACE2) is a component of the renin-angiotensin-aldosterone system, the most important regulator of vascular aging and the pathophysiology of hypertension [92]. In hypertension and related nephropathy, SIRT6 may also inhibit ACE2 expression in ECs [93], which requires further *in vivo* validation. The cardiovascular complications of SARS-CoV-2 infection include endothelial dysfunction and activation of immune cells, such as neutrophils [94,95]. Because SIRT6 represses the expression of the SARS-CoV-2 receptor ACE2 in ECs [93], aging-induced decreases in SIRT6 may be one of the mechanisms underlying SARS-CoV-2-induced vascular complications in aged populations.

VSMC senescence is an essential mechanism in vascular remodeling during the development of hypertension. A recent study demonstrated SIRT6 represses VSMC senescence [88]. This mechanism may also contribute to hypertension. The senescence of VSMCs and ECs is also functionally involved in hypertension and PAH. Thus, SIRT6 may also repress hypertension and PAH by maintaining VSMCs and ECs “young”.

These studies highlight the pivotal role of SIRT6 in aging-related cardiovascular disorders such as atherosclerosis and hypertension. The results also suggest that SIRT6 can prevent other types of vascular diseases, such as diabetic angiopathy, artery dissection, and ischemic vascular injury *via*a similar mechanism. Thus, SIRT6 is an ideal target for treating atherosclerosis and hypertension because the roles of SIRT6 in each type of vascular cell protect the vascular tissues.

### 4.2 Ischemic Stroke

Although recent studies have focused on SIRT6 in cardiovascular disorders, the role of SIRT6 in aging-related cerebrovascular diseases is not fully understood. Liberale et al. recently reported that endothelial SIRT6 preserved blood-brain barrier integrity and reduced stroke size by repressing endothelial apoptosis through AKT activation [96] (Fig. 3C). These findings improve the understanding of the role of SIRT6 in blood-brain barrier injury and stroke. Furthermore, preventing stroke by overexpressing SIRT6 is a promising translational strategy. Senescence-associated angiocrine factors from ECs are crucial for neuronal survival and post-stroke regeneration, which may be critical for SIRT6 function in stroke, as AKT controls the angiocrine phenotype. Another concern is how SIRT6 activates AKT ( *via* epigenetic regulation or not) and whether AKT is critically involved in SIRT6 function in ischemic stroke. A previous study of the heart revealed that SIRT6 repressed AKT [46], whereas Liberale et al. reported that SIRT6 activated AKT [96], suggesting that SIRT6 indirectly regulates AKT. Further studies are needed to determine the direct mechanism underlying SIRT6 function in stroke.

The mechanisms underlying SIRT6 functions in cardiovascular tissues significantly differ from those of its family member SIRT1. Generally, SIRT1 targets non-histone proteins in vascular cells, whereas SIRT6 regulates vascular cells *via* epigenetic mechanisms [26,50,53,55,88,90,97]. A recent follow-up study revealed that activated SIRT6 in macrophages repressed ischemic stroke by interacting with zeste homolog 2 to balance histone acetylation and methylation [97]. Endothelial SIRT6 may also function in ischemic stroke by directly regulating histone acetylation. In addition, VSMCs and immune cells express SIRT6, which is downregulated in the blood cells of patients with ischemic stroke [96]. Therefore, how SIRT6 regulates ischemic stroke *via*cell types other than ECs should be further evaluated.

## 5. SIRT6 Activators for Treating Cardiovascular Diseases

SIRT6 exerts anti-aging roles in vascular tissues, indicating the therapeutic potential of SIRT6 activators. Importantly, SIRT6 in all cell types can repress cardiovascular aging and diseases, suggesting SIRT6 as an ideal target for treating aging-related cardiovascular diseases. Therefore, studies are needed to identify SIRT6 activators. Several SIRT6 activators have been identified in recent years, with some candidates showing the therapeutic value (Table 3), as described in previous reviews [61,98,99].

Recent studies have provided a model for screening SIRT6 activators (e.g., MDL-800 and MDL-811) for treating cancer, ischemic stroke, and other aging-related diseases such as liver fibrosis. In 2018, Huang et al. identified MDL-800, MDL-801, and MDL-811 as SIRT6 activators that increased SIRT6 deacetylase activity by up to 22-fold *via*binding to an allosteric site [100]. MDL-800 and MDL-811 showed significant anti-cancer effects in hepatocellular carcinoma, non-small cell lung cancer, and colorectal cancer [100-102]. SIRT6 may also possess tumor-promoting functions; hence, some inhibitors have been developed [61,98,103]. Additionally, MDL-800 promoted deacetylation of lysine 54 on SMAD2 in hepatic stellate cells to alleviate liver fibrosis [104]. The novel SIRT6 activator MDL-811 ameliorated neuroinflammation and ischemic injury *via* the zeste homolog 2/FOXC1 axis [97], highlighting the value of MDL-811 for treating aging-related ischemic stroke. Notably, MDL-800 improves the genome stability and pluripotency of induced pluripotent stem cells derived from aged mice [72], revealing the potential of MDL-800 for preventing the senescence of stem cells and vascular cells.

Taken together, the currently identified SIRT6 activators MDL-800/811 show significant value as candidate drugs for treating aging and aging-related cardiovascular diseases. As SIRT6 controls the lifespan of non-human primates [63], the efficacy of MDL-800/MDL-811 for treating aging-related vascular disorders in non-human primates must be evaluated. More importantly, further studies are needed to examine the potential of SIRT6 activators such as MDL-800/MDL-811 for treating aging-related vascular diseases, such as atherosclerosis, hypertension, and stroke in humans, followed by preclinical and clinical trials.

**Table 3:** Identified SIRT6 activators.

Compound	EC50 (μM)	Max Activation	Substrate	Ref.
**MDL-800**	10	22-fold	H3K9ac	[100,104]
**MDL-801**	4.1	25.1-fold	H3K9myr	[129]
**MDL-811**	7.1	>15-fold	H3K9ac, H3K56ac	[97]
**Cyanidin**	460	55-fold	H3K9ac	[130]
**4H-chromen**	N/A	40-fold	H3K9ac	[131]
**Quercetin**	1200	2-fold	H3K9ac	[132]
**UBCS039**	38	3.5-fold	H3K9ac	[133]
**CL5D**	15	50-fold	H3K9ac	[134]
**Fucoidan**	N/A	335-fold	H3K9ac	[135]
**Oleoylethanolamide**	3.1	2-fold	H3K9ac	[136]
**Myristic acid**	246	35-fold	H3K9ac	[134]
**Nitro-fatty acid**	N/A	40-fold	H3K9myr	[137]
**12q**	0.58	>38-fold	Ac-RYQK(Ac)-AMC	[138]

N/A, not available.

## 6. Conclusion Remarks and Perspectives

SIRT6 is critical for mammalian development and determines the lifespan of rodents and non-human primates. Findings obtained in the past two years have highlighted the vital role of SIRT6 in preventing angiogenic defects, vascular diseases, atherosclerosis, hypertension, and ischemic stroke. The anti-senescence function is crucial for the protective roles of SIRT6 in aging and vascular diseases. Importantly, recent studies suggested the translational value of SIRT6 activators, such as MDL-800/811. However, some functions of SIRT6 need further addressing.

### (1) SIRT6-mediated metabolic regulation in vascular diseases

Recent studies have mainly focused on the function of SIRT6 in the epigenetic regulation of cell senescence and inflammation. Cardiovascular diseases are closely related to metabolic syndrome. Accumulating studies have shown that SIRT6 mediates liver and adipose metabolism [105,106], which may also contribute to the function of SIRT6 in vascular diseases. SIRT6 may regulate serum lipid and insulin sensitivity to prevent the development of age-related vascular disorders such as atherosclerosis and hypertension. The function of SIRT6 in regulating lifespan in mice and non-human primates largely depends on IGF signaling, a key regulator of cellular metabolism [62,63]. SIRT6 also prevents cardiac aging by epigenetically repressing the metabolic IGF-AKT pathway [46]. Furthermore, SIRT6-mediated metabolic changes in vascular cells may be equally critical in aging-related vascular injury [88]. Therefore, it is important to determine whether SIRT6 regulates the intracellular metabolism of vascular cells to prevent aging-related vascular diseases.

### (2) Insight into the mechanisms underlying SIRT6 inactivation

Increased methylation of the SIRT6 gene and decreased SIRT6 protein stability occur in vascular injury. Some studies suggested that SIRT6 is regulated by miRNAs and lncRNAs ( *e.g.*, miR-25-3p, LncRNA Blnc1, and LncRNA SNHG12) in vascular cells *in vitro* [107,108]; however, the mechanisms by which SIRT6 is repressed in aging and aging-related vascular diseases remain unclear. Further studies of the detailed mechanism underlying the aging-mediated decline in SIRT6 expression and activity will improve the understanding of SIRT6 and promote the design of SIRT6-targeted therapeutic translational strategies. The function and mechanisms of SIRT6 in aging-related vascular diseases such as myocardial infarction [109], arterial aneurysm [3], artery dissection [110], arterial calcification [111], and diabetic angiopathies [13] also require further analysis.

### (3) Vascular protective roles in aged animals and humans

SIRT6 is an anti-aging factor in rodents and primates [46,63,67]; therefore, it would be interesting to determine whether SIRT6 is critical for preventing vascular disorders in aged animals and humans. Our previous studies showed that SIRT6 expression is reduced in atherosclerotic plaques in patients [84]. In addition, polymorphisms in SIRT6 increase the risk of plaque burden and coronary artery disease [112,113]. Higher endothelial SIRT6 levels in plaques were also observed in patients administered GLP-1 receptor agonists. Notably, a clinical study implicated the low level of SIRT6 methylation (high SIRT6 expression) as a protective factor for Chinese longevity [67], and SIRT6 expression was decreased in cells of aged humans [65,66]. These findings reveal the critical role of SIRT6 in human aging and aging-related vascular diseases. Further studies of the role of SIRT6 in vascular diseases in aged human patients are needed to improve the understanding of SIRT6 and enable targeting of SIRT6 in aged patients with vascular diseases.
